# Glycosylation of Semi-Synthetic Isoflavene Phenoxodiol with a Recombinant Glycosyltransferase from *Micromonospora echinospora* ATCC 27932

**DOI:** 10.4014/jmb.2111.11032

**Published:** 2022-02-07

**Authors:** Minsuk Seo, Yurin Seol, Je Won Park

**Affiliations:** 1 Transdisciplinary Major in Learning Health Systems, Department of Integrated Biomedical and Life Sciences, Korea University, Seoul 02841, Republic of Korea; 2 Department of Integrated Biomedical and Life Sciences, Korea University, Seoul 02841, Republic of Korea; 3 School of Biosystems and Biomedical Sciences, Korea University, Seoul 02841, Republic of Korea

**Keywords:** MeUGT1, isoflavonoid glycosyltransferase, *Micromonospora echinospora*, phenoxodiol glucosides

## Abstract

Glycosyltransferase (GT)-specific degenerate PCR screening followed by in silico sequence analyses of the target clone was used to isolate a member of family1 GT-encoding genes from the established fosmid libraries of soil actinomycetes *Micromonospora echinospora* ATCC 27932. A recombinant MeUGT1 was heterologously expressed as a His-tagged protein in *E. coli*, and its enzymatic reaction with semi-synthetic phenoxodiol isoflavene (as a glycosyl acceptor) and uridine diphosphate-glucose (as a glycosyl donor) created two different glycol-attached products, thus revealing that MeUGT1 functions as an isoflavonoid glycosyltransferase with regional flexibility. Chromatographic separation of product glycosides followed by the instrumental analyses, clearly confirmed these previously unprecedented glycosides as phenoxodiol-4'-α-*O*-glucoside and phenoxodiol-7-α-*O*-glucoside, respectively. The antioxidant activities of the above glycosides are almost the same as that of parental phenoxodiol, whereas their anti-proliferative activities are all superior to that of cisplatin (the most common platinum chemotherapy drug) against two human carcinoma cells, ovarian SKOV-3 and prostate DU-145. In addition, they are more water-soluble than their parental aglycone, as well as remaining intractable to the simulated in vitro digestion test, hence demonstrating the pharmacological potential for the enhanced bio-accessibility of phenoxodiol glycosides. This is the first report on the microbial enzymatic biosynthesis of phenoxodiol glucosides.

## Introduction

**Fig. 1:**
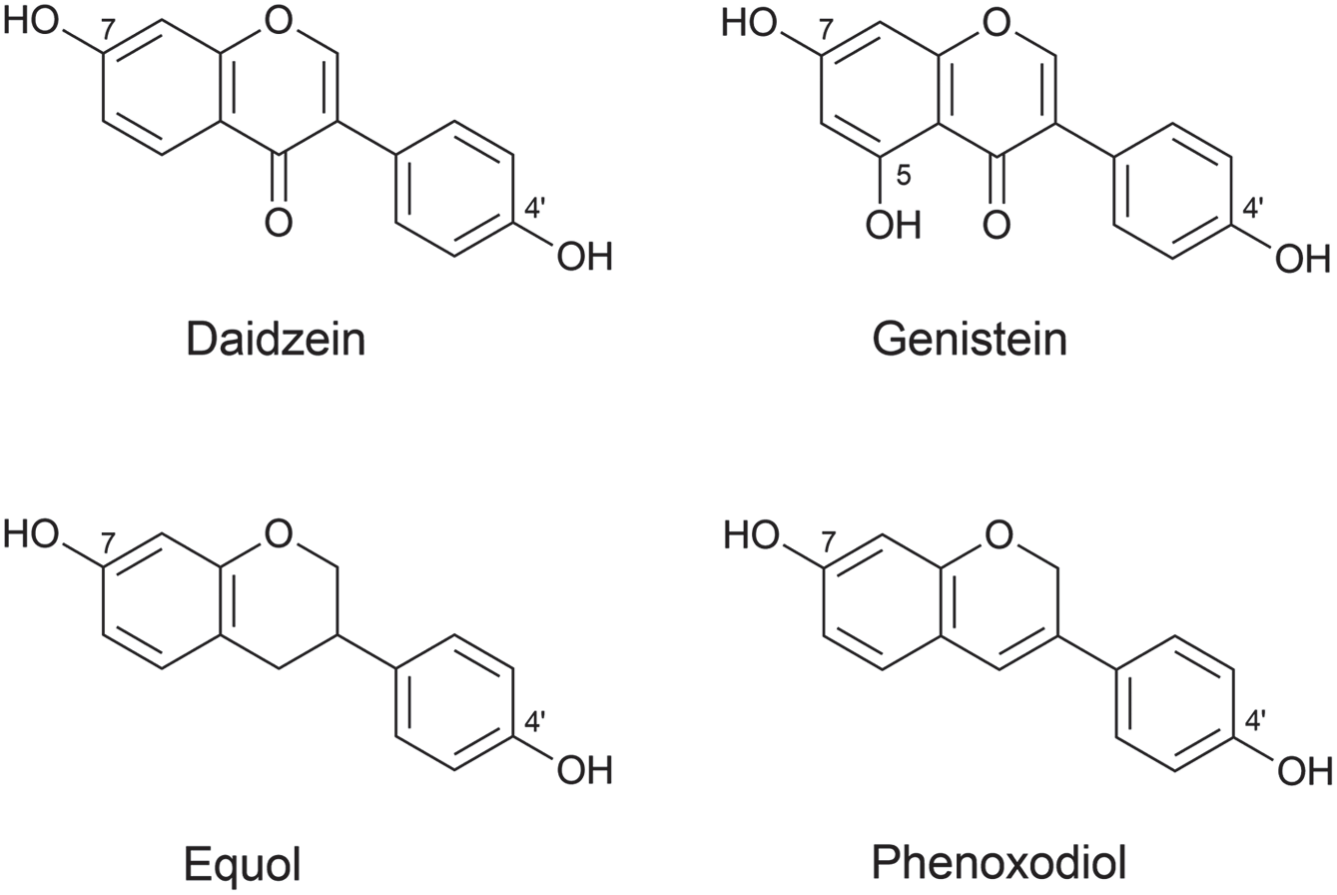
Chemical structures of isoflavonoids described in this study.

In conjunction with the global trend of population aging, cancer is still being recognized as one of the leading causes of death worldwide, accounting for nearly 10 million deaths in 2020. Therefore, the development of novel anticancer agents has become a pressing issue. Flavonoids, which have attracted considerable attention as a main ingredient in functional health foods, represent as a series of polyphenolic secondary metabolites (with C6-C3-C6 carbon skeleton) derived mostly from plants [1]. Isoflavonoids are a subclass of flavonoids with extensive pharmacological activities and exceedingly low toxicity and have therefore become a hotspot of new drug discovery. They possess a wide range of biologically beneficial properties such as antioxidant, anti-inflammatory, anti-viral, anti-aging, and anti-cancer activities. Moreover, their dietary intake has been shown to exhibit beneficial anti-obesity effects on human metabolism, and may also be helpful for relieving menopausal symptoms in postmenopausal women [2, 3]. Among them, daidzein (4',7-dihydroxyisoflavone) and genistein (4',5,7-trihydroxyisoflavone) (Fig. 1) are known to show physiological activities against mammalian cells, such as controlling cell cycle progression, mitotic arrest, or apoptosis [4, 5]. In particular, genistein is known to have low toxicity towards normal mammalian cells and tissues, while displaying effective anti-cancer activities against various types of cancer such as ovarian, prostate, and gastric cancers [5]. However, both the limited bioavailability of genistein and its diverse metabolism make it difficult to maintain proper plasma concentration when administered in vivo, which ultimately created problems with clinical applicability [6]. Meanwhile, equol (4',7-isoflavandiol) (Fig. 1) is an isoflavone metabolized from daidzein by intestinal microbiota in mammals and is referred to as non-steroidal estrogen with structural similarities to human hormone estrogen. Accordingly, equol shows biological activities, including prevention of prostate cancer, as well as alleviation of menopausal symptoms, male pattern baldness, and acne [7]. On the other hand, phenoxodiol (also known as idronoxil or 4',7-dihydroxyisoflav-3-ene) (Fig. 1), is an isoflavene analog as a semi-synthetic derivative of genistein and shows promising anti-proliferative activities on a broad range of human cancer cell lines, thus having been assessed in clinical trials for drug-resistant ovarian and hormone-refractory prostate cancer [8]. This investigational agent also inhibits DNA topoisomerase II by stabilizing the cleavable complex, thereby preventing DNA replication and resulting in tumor cell death [8].

The biosynthesis of isoflavonoids has long been considered one of the most characteristic natural product biosynthetic pathways in plants. However, their presence among diverse microbial sources including fungi and actinomycetes has been recently reported in studies, thus supporting the feasibility of microbial biotransformation (or structural reconstruction) of isoflavonoids [9, 10]. Glycosylated flavonoids possessing biological activities, stability, and solubility, have been found more effective than their parental aglycones [11, 12]. Enhancement of pharmacological activities and development of pharmaceutical candidates through structural modifications to the parental molecules are recognized as influential tools for the development of incrementally modified drugs, *e.g.*, the enzymatic biosynthesis of glycosides using glycosyltransferases (GTs) capable of transferring sugar molecules to hydroxyl function within the target molecules (or aglycones). Such glycosylation can improve the solubility and bio-absorbability of the glycosylated ones, more so than the parental aglycones, which will surely be helpful in reformulating and developing new formulations of drugs and turning them into prodrugs [12, 13]. Moreover, it is extremely hard to create glycosides using currently available chemical synthetic tools. Indeed, phenoxodiol could be further reconstructed through molecular hybridization to enhance its anti-cancer activity. In 2013, it was shown that phenoxodiol was hybridized with a 1-amino-2-propanol moiety within the b-blocker propranolol, leading to hybrids with enhanced anti-proliferative and anti-angiogenic activities [14]. A series of phenoxodiol-thiosemicarbazone hybrids were also recently synthesized via the condensation reaction and displayed potent anti-proliferative and superior selectivity against cancer cells over normal cells, thus clearly supporting molecular reconstruction of phenoxodiol as an efficacious strategy to further promote its pharmacological potential [15]. Herein, we aimed to isolate an isoflavonoid GT-encoding gene, from the formerly established fosmid libraries of soil actinomycetes *Micromonospora echinospora* (former *rhodorangea*) ATCC 27932 [16, 17], by employing GT-specific degenerate PCR. Next, this glycosyltransferase MeUGT1 was heterologously expressed in *E. coli* BL21(DE3) as a His-tagged recombinant protein. In addition, the biosynthesis of new phenoxodiol glycosides, whose chemical structures are unprecedented, was carried out through the enzymatic reaction of the recombinant MeUGT1 together with substrates (including phenoxodiol aglycone and nucleotidyl-activated sugar), and their biological activities together with physicochemical properties were comparatively evaluated.

## Materials and Methods

### Chemicals and Reagents

Phenoxodiol, equol-4'-*O*-α-glucoside, equol-7-*O*-α-glucoside and genistin β-glucoside were purchased from Sigma-Aldrich (USA), whereas daidzin β-glucoside was from TCI Research Chemicals (Japan). Isopropyl β-D-1-thiogalactopyranoside (IPTG), 3-(4,5-dimethylthiazol-2-yl)-2,5-diphenyltetrazolium bromide (MTT), L-ascorbic acid, pepsin, pancreatin and bile salts were obtained from Sigma-Aldrich, while cisplatin was from Roche Diagnostics (Germany). The others were of reagent grade.

### Recombinant MeUGT1 and Its Enzymatic Reaction

Degenerate PCR screening was used to isolate a GT-encoding gene from the fosmid libraries of a geneticin aminoglycoside-producing *M. echinospora* ATCC 27932, and an orf89 (designated with NdeI and XhoI cut) was amplified by PCR. This NdeI/XhoI-digested fragment excised from the subcloned pGEM T-Easy vector was cloned into the same sites of the protein expression vector pET-28a(+) (Novagen, USA) and expressed as an N-terminal fusion protein with a hexa-histidine (His-6) tag in *E. coli* BL21(DE3) strain. After induction using 0.5 mM IPTG (Sigma-Aldrich) followed by an overnight incubation at 22°C, the cells were harvested and then disrupted by ultrasonication. A His-tagged protein was purified with TALON Metal Affinity Resin (Clontech, USA) conditioned with 50 mM phosphate buffer (0.3 M NaCl, 20 mM imidazole) and incubated at 4°C for 1 h. After 5 min of refrigerated centrifugation at 2,000 ×*g*, the resin was introduced into a disposable column and washed with phosphate buffer containing 20 mM imidazole, equivalent to 10 times the amount of the resin. Finally, recombinant MeUGT1 bound to the above resin was purified with 3 ml of phosphate buffer containing 220 mM imidazole.

For the in vitro reactions, phenoxodiol (Sigma-Aldrich) was dissolved in methanol at 50 mM and diluted in a reaction buffer (50 mM phosphate buffer, 10 mM magnesium chloride, 1 mg/ml bovine serum albumin) to a final concentration of 1 mM. Then, 30 µM of recombinant MeUGT1, along with 2 mM of UDP-Glc as a glycosyl donor, was added and incubated at 37°C for 1 hr to examine the GT activity of MeUGT1 on semi-synthetic phenoxodiol aglycone. An equal amount of ethyl acetate was immediately added to quench the reaction. The separative organic solvent layer was evaporated to dryness by vacuum centrifugation, and then the extracts were reconstituted in methanol prior to HPLC-tandem mass spectrometric (MS/MS) analysis.

### Instrumental Analyses

HPLC-LCQ ion-trap MS/MS (ThermoFinnigan, USA) was performed using both acetonitrile:methanol:water:formic acid (40:40:19.8:0.2 v/v/v/v) mixture as isocratic mobile phase at a flow rate of 150 µl/min, and Acquity CSH C_18_ reversed-phase column (Waters, 50 × 1.0 mm, 1.7 µm; USA) as the stationary phase. The column effluent was introduced into the MS/MS operated in the positive ion mode. Acquisition was performed using MS/MS operated in the selective reaction monitoring mode by choosing a pair of mass transitions specific to phenoxodiol and the corresponding glycosides to detect the transition of the protonated precursor ion to the dominant product ion (241.2 [M+H]^+^ > 223.2 [M-H_2_O+H]^+^ as a dehydrated product ion for phenoxodiol and 403.4 > 241.2 [M-Glc+H]^+^ as an aglycone product ion for phenoxodiol glycoside). In addition, both high-resolution (HR) LCT-premier XE MS (Waters) and Varian INOVA 500 nuclear magnetic resonance (NMR, Varian Inc., USA) were employed for the structural elucidation of two kinds of phenoxodiol glycosides.

### Bioactive and Physicochemical Assays

DPPH (1,1-diphenyl-2-picryl-hydrazyl-hyate) free radical scavenging assay (absorbance at 515 nm in methanol) was employed for determining the in vitro antioxidant activity of phenoxodiol and its generated glycosides; L-ascorboic acid (Sigma-Aldrich) utilized as a positive control, and antioxidant activity calculated as IC_50_ value (as the concentration of an antioxidant at which 50% inhibition of free radicals occurs). MTT-based, anti-proliferative activities of phenoxodiol and its glycosides against two cancer cell lines (SKOV-3 human ovarian carcinoma and DU-145 human prostate carcinoma cells) were in vitro examined with colorimetric absorbance at 570 nm; cisplatin (Roche Diagnostics) utilized as a positive control, and cytotoxic activity measured as IC_50_ value (as the concentration of a sample [dissolved in 20% DMSO] that induces 50% reduction in target cell viability).

The physicochemical properties of phenoxodiol aglycone and its two glycosides were compared as follows: to determine the water solubility, each dried compound (weighed at 0.1 mg) was vigorously vortexed with equal volume (200 µl) of distilled water and ethyl acetate, then centrifuged at 3,000 ×*g* for 5 min. Aliquots (50 µl) were removed from each separative layer, and then subjected to HPLC-MS/MS analysis as reported before [18]. The partitional existence of a total three compounds extracted from each layer was determined as a relative percentage. Finally, the simulated in vitro digestion was carried out according to the previous publication [19] with slight modifications. Phenoxodiol glycosides (each 0.2 mg) were dissolved in 2 ml of distilled water together with 500 μl of α-amylase-CaCl_2_ solution, then incubated at 37°C for 10 min. After the prompt addition of 10 mg of pepsin (14,800 U; Sigma-Aldrich) and subsequent pH adjustment to 2.0, each gastric lysate was incubated at 37°C for 1 h. Next, the pH was increased up to 6.5, followed by the addition of 0.5 ml of pancreatin (4 µg/ml, Sigma-Aldrich) and bile salts solution (25 µg/ml dissolved in 0.1 mM NaHCO_3_; Sigma-Aldrich). The pH of this digestion was then adjusted to pH 7.4 and incubated at 37°C for 2 h. Each gastrointestinal lysate was centrifuged after the pH was adjusted to 2.0, and then the supernatants were extracted with acetonitrile. An aliquot (200 µl) was directly subjected to HPLC-MS/MS analysis; both genistin β-glucoside (Sigma-Aldrich) and daidzin β-glucoside (TCI Research Chemicals, Japan) were utilized as controls for the plant-origin, β-type isoflavonoid glycoside.

## Results and Discussion

In our previous report [16], we employed degenerate PCR screening using the fosmid libraries of a geneticin aminoglycoside-producing *M. echinospora* ATCC 27932, and a total of six different kinds of amplified clones were chosen and then thoroughly sequenced: two (orf112 and orf123) were found to be involved in geneticin aminoglycoside biosynthesis by sequence alignment using BLAST (http://www.ncbi.nih.gov/BLAST); one (orf74) was further characterized as a uridine diphosphate-glucose (UDP-Glc) sterol glycosyltransferase (MrSGT deposited under GenBank accession no. KT98252); the remaining three (orf89, orf132 and orf173) surely encode family 1 GT, but are supposed to act on different kinds of aglycones. Hence, an orf89 (designated with NdeI and XhoI cut) was amplified by PCR, and its product was 1,242 bp in length. The gene encodes a predicted 44.8-kDa protein (annotated as MeUGT1 and deposited under GenBank accession no. MZ772034). In silico BLAST analysis of the deduced protein revealed dominant homology with GTs from *M. rosaria* (58%), *M. carbonacea* (44%), and *Bacillus licheniformis* (44 and 42%). The first three GTs are still putative proteins without any proof of functional characterization, whilst the last one, YjiC, has been extensively utilized for the glycosylation of small molecules including flavonoids and macrocyclic polyketides [20, 21]. Furthermore, the presence of a significant conserved motif (as nucleotide-sugar donor binding domain) in the C-terminal region supports that the target orf89 product is a member of family 1 GTs.

**Fig. 2:**
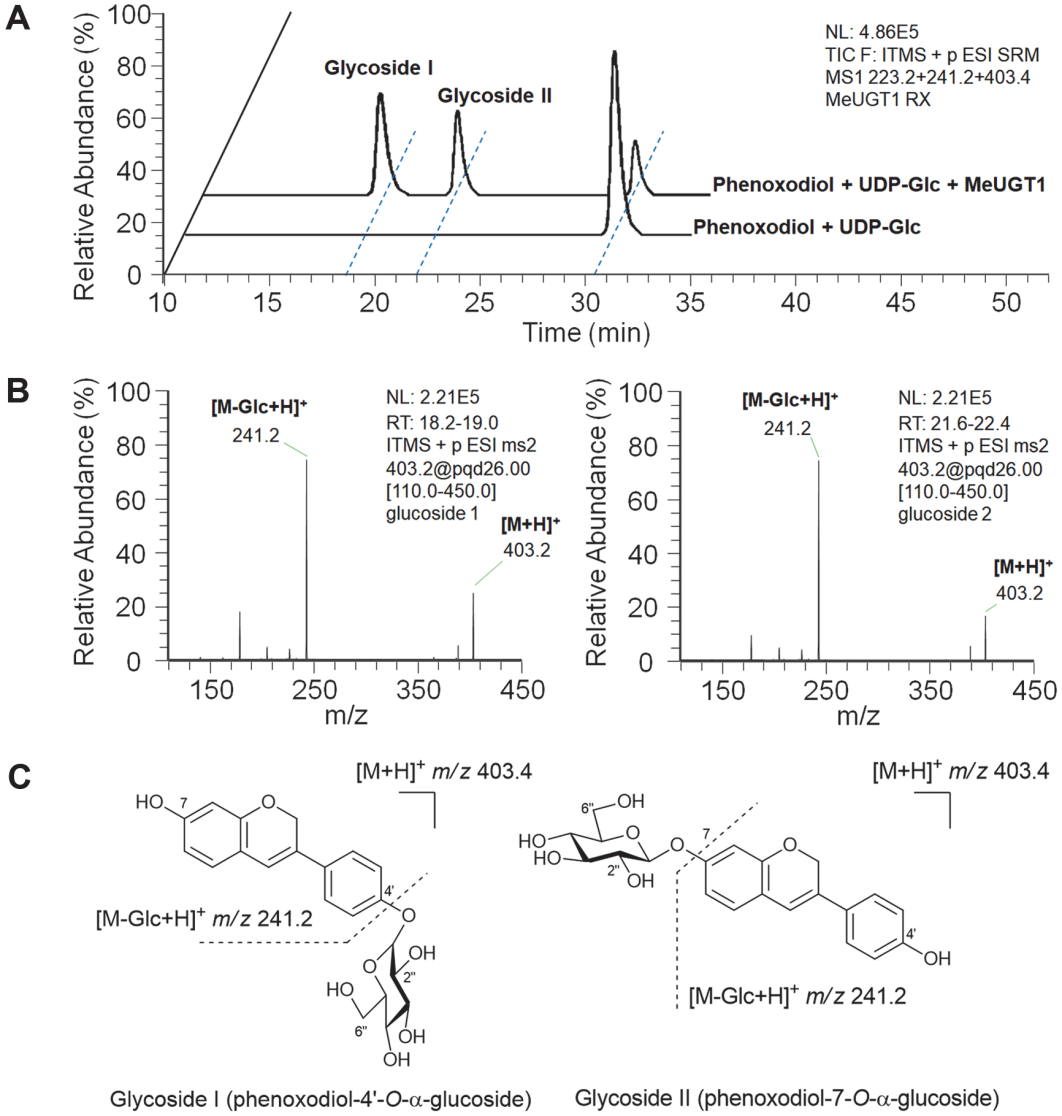
HPLC-MS/MS analyses of the in vitro enzymatic reactions of MeUGT1 with phenoxodiol and UDPGlc. (**A**) Mass chromatogram and (**B**) MS/MS spectra obtained, and (**C**) the observed fragmentation patterns of the expected phenoxodiol glucosides.

The in vitro enzymatic formation of the corresponding phenoxodiol glycosides was detected using HPLC-MS/MS analyses, and the MS fragmentation patterns (*i.e.*, glycone molecular ion, aglycone product ion) characteristic of the product phenoxodiol glycosides clearly indicated the attachment of a glycosyl moiety onto the phenoxodiol isoflavene, representing them as a series of phenoxodiol-*O*-glucoside (Fig. 2). On the HPLC-MS/MS chromatogram obtained from the reactant, in which UDP-Glc was solely supplemented as the glycosyl donor, two peaks of glycosylated products were separately detected, thus possibly indicating dual attachment of Glc moiety, with different positions at C4' and C7, onto the phenoxodiol aglycone; the conversion ratio of glycosides from the phenoxodiol aglycone was estimated to be about 0.5 and 0.3, for glycoside I and II, respectively (Fig. 2). Meanwhile, the CombiFlash Rf MPLC system (Teledyne ISCO, USA) was utilized to isolate and purify enough quantities of phenoxodiol glycosides to elucidate their structural features. After multiple scale-up reactions (>10 batches of reactants) followed by MPLC chromatographic separation, two phenoxodiol-*O*-glucosides, which were fractionated as separative peaks with different retention times (glycoside I at 18.6 min of retention and glycoside II at 22.0 min of retention; see Fig. 2), were obtained as a white powder (4.4 and 3.8 mg each), respectively. High-resolution (HR) LCT-premier XE MS (Waters) analysis of these phenoxodiol-*O*-glucosides gave an identical *m/z* of 403.3982 consistent with the derived protonated formula of C_21_H_22_O_8_ (403.3946). The ^13^C-NMR spectra of glycosides I and II displayed fifteen aglycone carbon signals and six glucose carbon signals clearly, and the appearance of proton peaks representing glucose moieties appeared within the range of *δ_H_* 3.22 to 5.37 and confirmed that each glycoside contains a glucose moiety attached to phenoxodiol aglycone (see Table S1). ^1^H-NMR spectra of both glycosides exhibited an anomeric proton signal at *δ_H_* 5.87, and the presence of up-field shifts due to the locational glycosylation of aglycone indicated that the chemical structures of the two glycosides could be designated as phenoxodiol-4'-*O*-glucoside and phenoxodiol-7-*O*-glucoside, respectively (Table S1). The anomeric configuration (α- or β-) of the attached glycosyl moiety was further confirmed by listed chemical shifts of NMR spectra. As authentic standards for the above glycosides, equol-4'-*O*-α-glucoside and equol-7-*O*-α-glucoside were favorably purchased from Sigma-Aldrich. The anomeric proton signal at *δ_H_* 5.87 found in all purified compounds indicated the equatorial (α) resonance (instead of the up-fielded axial one), and which is also consistent with that of the authentic standards. Moreover, a coupling constant (*J*) of 2.6 to 2.7 Hz detected at the anomeric proton of both glucosides was assigned an apparent α-anomer, thus phenoxodiol glycosides I and II were consequently elucidated as phenoxodiol-4'-*O*-α-glucoside and phenoxodiol-7-*O*-α-glucoside, respectively (Table S1). Further in vitro enzymatic reactions were examined using the above glucosides as a substrate of MeUGT1, instead of phenoxodiol aglycone, to examine whether dual glycosylation (at both 4' and 7 positions) occurs or not. However, throughout the overnight reaction, we were not able to trace any phenoxodiol bis-glycosides as products in the reactant, thus indicating the regio-specificity (or regio-selectivity) of MeUGT1 for the attachment of a glucose moiety onto a phenoxodiol substrate.

**Fig. 3:**
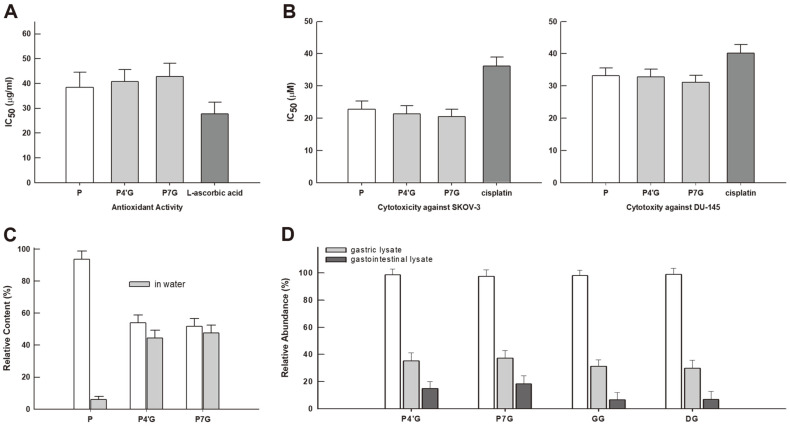
Biological activities and physicochemical properties of phenoxodiol and its two glucosides. (**A**) Antioxidant activity (L-ascorbic acid as a positive control), (**B**) anti-proliferative activity against two human carcinoma cell lines (cisplatin as a positive control), (**C**) water solubility, and (**D**) bio-accessibility against the simulated in vitro digestion model (genistin β-glucoside and daidzin β-glucoside as comparative ones). P: phenoxodiol, P4'G: phenoxodiol-4'-*O*-α- glucoside, P7G: phenoxodiol-7-*O*-α-glucoside, GG: genistin β-glucoside, DG: daidzin β-glucoside.

After elucidating the structural features of both of the obtained phenoxodiol glycosides, their biological activities (*i.e.*, antioxidant and anti-proliferative activity) together with their physicochemical properties (*i.e.*, water solubility and stability against in vitro gastrointestinal digestion model) were evaluated by comparison with those of the parental aglycone. In a DPPH assay of antioxidant activity, two glycosides were shown to be indifferent from their parental phenoxodiol aglycone, while the free radical scavenging activities of the above three compounds were all inferior to that obtained from positive control L-ascorboic acid (Fig. 3A). Next, the cytotoxicity of phenoxodiol glycosides against two carcinoma cell lines was examined by comparison with their parental aglycone. IC_50_ values of glycosides against SKOV-3 cells averaged 20.0 to 23.1 µM, a range which is comparable to that of phenoxodiol (22.8 µM). As expected, the IC_50_ values of glycosides against DU-145 cells were also in the range of 31 to 32.8 µM, which is analogous to that of phenoxodiol (33.1 µM). Moreover, according to the obtained IC_50_ values, the anti-proliferative activities of phenoxodiol and their glycosides were all superior to cisplatin as an existing chemotherapy agent (Fig. 3B). Therefore, based on the results from paired in vitro biological activities carried out herein, it was clearly determined that the structural modification (*i.e.*, glycosylation) onto the isoflavene phenoxodiol does not attenuate the parental activities, thus displaying the expandability of the pharmacological potential of the semi-synthetic isoflavene phenoxodiol. On the other hand, the physicochemical properties of phenoxodiol aglycone and its two glycosides were further compared. In the water solubility test, both phenoxodiol glycosides appear to be more water-soluble than their parental aglycone; phenoxodiol was partitioned in water at 5.9%, whereas both glycosides were partitioned within the range from 44.6 to 47.8% (Fig. 3C). Besides, when we compared the chemical stabilities (or gastrointestinal fate) of phenoxodiol glycosides and two β-type isoflavonoid glycosides against the simulated digestion model, phenoxodiol glycosides (above all, phenoxodiol-7-*O*-α-glucoside) seem to be more resistant to digestive processes; α-type phenoxodiol glycosides appeared to retain their chemical features (14.8 to 18.4%) even after the simulated digestion model, while β-type isoflavonoid glycosides reserved their own structures below 7% (Fig. 3D). Many isoflavonoid glycosides of plant origin are liable to β-glucosidase activity existing in the small intestine, owing to their innate β-anomeric configuration [7,22]. However, phenoxodiol glycosides, which are biosynthesized as α-anomers, are likely to be insusceptible to the β-linked glycolytic digestion derived from pancreatin enzyme mixtures. Indeed, the chemical fate of aglycone, derived from glycosides, in the simulated gastric and intestinal digesta allows us to comprehend the bio-accessibility of the glycoside of interest.

Taken together, the enzymatic function of MeUGT1 originating from *M. echinospora* is proven as an isoflavonoid glycosyltransferase, which utilizes UDP-Glc as a glycosyl donor for the attachment of this sugar moiety on the substrate phenoxodiol aglycone. The presence of two products derived from the recombinant GT reaction revealed that the appendage of glucose onto the substrate occurs at different (4' and 7) hydroxyl groups onto phenoxodiol, thus suggesting MeUGT1 as a GT with regional flexibility. According to the chromatographic separation of product glycosides followed by the instrumental analyses, the anomeric configuration of the attached glycosyl moiety was further proven as α-configuration. Therefore, these unprecedented glycosides are phenoxodiol-4'-α-*O*-glucoside and phenoxodiol-7-α-*O*-glucoside, respectively. The antioxidant activities of the above glycosides are almost equivalent to that of phenoxodiol aglycone, whereas their anti-proliferative activities are superior to that of cisplatin (the most common platinum chemotherapy drug) against two human carcinoma cell lines, ovarian SKOV-3 and prostate DU-145. Moreover, as for physicochemical properties, the two glycosides are more water-soluble than their parental phenoxodiol, as well as being more resistant in the simulated in vitro digestion model, hence indicating the possibility for the enhanced bio-accessibility of phenoxodiol glycosides. Further studies on both the GT kinetics of MeUGT1 on other related substrates and its introduction into microbial cell factory for the in vivo production of target glycosides could be carried out, and the resulting biosynthetic new glycosides might contribute to the glyco-expansibility of structural diversity of isoflavonoids with improved pharmacological potential.

## Supplemental Materials

Supplementary data for this paper are available on-line only at http://jmb.or.kr.
