# Supplementary material for: Glycosylation of Semi-Synthetic Isoflavene Phenoxodiol with a Recombinant Glycosyltransferase from Micromonospora echinospora ATCC 27932
Source: J Microbiol Biotechnol. 2022 Feb 7;32(5):657–62. doi: 10.4014/jmb.2111.11032 (PMC9628886; doi:10.4014/jmb.2111.11032)
Supplement: Supplementary file 1 [file jmb-32-5-657-supple.pdf]

J. Microbiol. Biotechnol.
https://doi.org/10.4014/jmb.2201.01023

# Supplementary Materials

## Glycosylation of semi-synthetic isoflavene phenoxodiol with a recombinant glycosyltransferase from *Micromonospora echinospora* ATCC 27932

**Supplementary Table 1.** $^{1}$H- and $^{13}$C-NMR data (500 MHz; DMSO-$d_6$) of phenoxodiol-4'-*O*-α-glucoside and phenoxodiol-7-*O*-α-glucoside, compared with those of phenoxodiol aglycone.

| | phenoxodiol-4'-*O*-α-glucoside | | phenoxodiol-7-*O*-α-glucoside | | phenoxodiol | |
|---|---|---|---|---|---|---|
| | $^{1}$H (ppm) | $^{13}$C (ppm) | $^{1}$H (ppm) | $^{13}$C (ppm) | $^{1}$H (ppm) | $^{13}$C (ppm) |
| Aglycone | | | | | | |
| 1 | 4.60 | 68.3 | 4.60 | 68.2 | 4.60 | 68.3 |
| 2 | | 126.2 | | 126.2 | | 126.1 |
| 3 | 7.22 *s* | 127.1 | 7.21 *s* | 127.3 | 7.22 *s* | 127.3 |
| 4 | | 117.1 | | 116.9 | | 117.2 |
| 5 | 8.08 | 130.8 | 8.15 | 130.7 | 8.06 | 130.7 |
| 6 | 6.24 | 108.0 | 6.59 | 110.6 | 6.26 | 107.9 |
| 7 | | 158.4 | | 158.9 | | 158.4 |
| 8 | 6.58 *s* | 101.9 | 6.64 *s* | 101.0 | 6.55 *s* | 101.6 |
| 9 | | 155.3 | | 154.9 | | 155.0 |
| 7-OH | 5.35 | | | | 5.34 | |
| 1' | | 131.4 | | 132.0 | | 132.1 |
| 2' | 7.29 | 129.0 | 7.30 | 129.8 | 7.31 | 129.8 |
| 3' | 6.92 | 114.0 | 6.69 | 115.3 | 6.69 | 115.2 |
| 4' | | 156.1 | | 157.9 | | 157.9 |
| 5' | 6.77 | 114.5 | 6.75 | 114.7 | 6.74 | 114.7 |
| 6' | 7.30 | 129.6 | 7.33 | 129.8 | 7.33 | 129.5 |
| 4'-OH | | | 5.30 | | 5.31 | |
| Glucose | | | | | | |
| 1" | 5.87 *d* (2.7) | 109.2 | 5.87 *d* (2.6) | 109.1 | | |
| 2" | 3.48 *m* | 73.3 | 3.48 *m* | 73.4 | | |
| 3" | 3.37 *dd* | 76.7 | 3.36 *dd* | 76.7 | | |
| 4" | 3.23 *dd* | 71.4 | 3.22 *dd* | 71.2 | | |
| 5" | 3.46 *m* | 81.6 | 3.44 *m* | 81.3 | | |
| 6" | 3.67 *d* | 62.0 | 3.66 *d* | 62.1 | | |
| | 3.51 *m* | | 3.51 *m* | | | |

Coupling constant (*J* in Hz) in parentheses
